# ID 337 - Impacto Econômico das Doenças Cardiovasculares no Brasil: projeções de custos diretos e indiretos para os próximos 5 anos

**DOI:** 10.5327/2237-9622.2025.v34s1.337

**Published:** 2025-11-25

**Authors:** Alessandro Bigoni, Alessandro Bigoni, Ione Oliveira, Lucas Tôrres, Ana Carolina Padula, Lucas Fahham

**Keywords:** doença cardiovascular, sistema de saúde, custos diretos, custos indiretos

## Abstract

**Introdução:**

As doenças cardiovasculares (DCV) são a principal causa de óbito no Brasil, superando neoplasias e doenças respiratórias entre 2015 e 2019. A doença aterosclerótica (DA), responsável por 67% dessas mortes, inclui condições como infarto agudo do miocárdio (IAM) e acidente vascular encefálico isquêmico (AVCI), com recorrência hospitalar superior a 20% no primeiro ano pós-evento, impactando a economia e a sociedade. Em relação aos custos diretos e indiretos das DCV no período de 2010-2015, Siqueira . (2017) estimaram um total de R$ 37 bilhões. Diante do envelhecimento populacional que amplia casos e gastos em saúde, este estudo visa estimar os custos diretos e indiretos relacionados à DA no Brasil nos próximos cinco anos.

**Método:**

Este estudo estimou o uso de recursos em saúde e custos, utilizando dados do Ministério da Saúde (MS) e da Agência Nacional de Saúde Suplementar (ANS) para o período de 2019 a 2024. As doenças cardiovasculares associadas à DA incluíram: doenças isquêmicas do coração (DIC), acidente vascular isquêmico (AVI) e doença arterial periférica (DAP). Os custos diretos foram calculados multiplicando-se o número de eventos por seus respectivos valores nas tabelas da Terminologia Unificada em Saúde Suplementar (Tuss) e do Sistema de Gerenciamento da Tabela de Procedimentos, Medicamentos e Órteses, Próteses e Materiais Especiais do SUS (Sigtap). Os custos indiretos consideraram óbitos e períodos médios de afastamento do trabalho, com base em estimativas da Pesquisa Nacional por Amostra de Domicílios para a população economicamente ativa (14 a 64 anos). Cada óbito foi multiplicado pelo tempo de atividade perdido e rendimento médio anual. As internações foram multiplicadas pelo tempo médio de recuperação e rendimento médio mensal. As projeções utilizaram o método de (ETS), comumente utilizado em séries temporais não estacionárias.

**Resultados:**

Entre os anos de 2024 e 2029, estima-se, em média, mais de 553 mil internações anuais no SUS, totalizando um custo de R$ 2,4 bilhões por ano, com um custo médio de R$ 4,3 mil por internação. Na saúde suplementar, projetam-se mais de 129 mil internações anuais, totalizando R$ 2,8 bilhões por ano e um custo médio de R$ 22,3 mil por internação. Eventos cardiovasculares associados à DA devem causar cerca de 35,9 mil óbitos em 2025, aumentando para 36,4 mil em 2029, gerando uma carga média de R$ 4,3 bilhões em custos indiretos por óbitos. Os custos indiretos por absenteísmo somarão, em média, R$ 181 milhões por ano. Assim, as doenças cardiovasculares associadas à DA deverão custar mais de R$ 49 bilhões ao sistema de saúde em cinco anos.

**Conclusão:**

Além de afetarem a qualidade de vida, as DCV impõem um pesado fardo econômico no sistema de saúde. Por exemplo, no SUS, as intervenções coronárias percutâneas dobraram entre 2008 e 2022. Na saúde suplementar, a internação por acidente vascular encefálico pode custar até R$ 65 mil, enquanto a angioplastia com pode superar R$ 37 mil (Teich ., 2015). Uma limitação deste estudo é a potencial subestimação dos custos e da utilização de recursos devido à subnotificação de casos nas bases de dados. Nossos resultados indicam um aumento futuro nos procedimentos e nos gastos com DA, projetados a superar R$ 49 bilhões nos próximos cinco anos. Diante do envelhecimento da população brasileira e do aumento da expectativa de vida, são necessárias intervenções preventivas eficazes, políticas de saúde direcionadas e um melhor controle dos fatores de risco para mitigar os impactos econômicos.

